# Supplementary material for: English version of the Computer Vision Symptom Scale (CVSS17): Translation and Rasch analysis-based cultural adaptation
Source: PLoS One. 2025 Apr 16;20(4):e0316936. doi: 10.1371/journal.pone.0316936 (PMC12002468; doi:10.1371/journal.pone.0316936)
Supplement: S1 File — The name and role of each participant are provided, along with a summary of the discrepancies discussed in the session. Summary statistics for the Rasch analysis conducted on the pre-test responses are also displayed. (PDF) [file pone.0316936.s001.pdf]

Expert commitee report

Membership:

Role	Name:
Methodologist	Rosario Susi
Clinician#1	Mark Rosenfield
Clinician#2	Mariano González
Clinician#3	Kathleen Hoang
Translator #1	Adrian Burton
Translator #2	SUNY staff (couldn't come)
Back Translator #1	Ana Burton
Back Translator #2	Mariano González

Report of discrepancies:

**Issue: (specify item # and describe issue)**
Instructions1: We use the simple past, but back-translations are expressed in simple present
Instructions2: Is it clear?
Instructions3: Shall we use other word rather than "circle"? Shall we note that there's no right or better answers?
A2: Is it clear that we didn't try to say "are blurry" or "seem Blurry"?
A9: We use the simple past, but back-translations are expressed in simple present
A20: Is better using "a lot" instead of "very much"
A22. Is there any other expression rather than "strain to see well"?
A28: Simple past and alternatives to "feel like you're crossing your eyes?
B7 and B8: Alternatives?
C23: Alternatives to "relieve"?
response categories2: A few time, Part of the time and Much time?
response categories3: Constantly?
Response categories: Many times and lots of times?
Response categories: "Somewhat" instead of "quite"

Potential issues (highlighted in red):

CVSS17 Questionnaire	CVSS17 Direct Translation	CVSS17 Back-Translation #1	CVSS17 Back-Translation #2
Original Version Item	DT Item	BT1 ITEM	BT2 ITEM
Instructions: LAS PREGUNTAS QUE SIGUEN SE REFIEREN A CÓMO SE HA SENTIDO DURANTE SUS ÚLTIMAS CUATRO SEMANAS DE TRABAJO	Instructions: FOLLOWING QUESTIONS ASK ABOUT HOW YOU FELT DURING YOUR LAST FOUR WORKING WEEKS:	Instructions: Las siguientes preguntas son acerca de como se siente durante las ultimas cuatro semanas de trabajo	Instructions: LAS SIGUENTES PREGUNTAS SE REFIEREN A LAS ULTIMAS CUATRO SEMANAS DE TRABAJO
Si usa gafas o lentes de contacto habitualmente en su trabajo, por favor responda a todas las preguntas pensando en cómo se siente cuando sí las lleva puestas	If you normally wear glasses or contact lenses during most of your working hours, answer as if you were wearing them	Si normalmente usa espejuelos o lentes de contacto durante la mayoria de las horas de trabajo, responda si los ha estado usando.	Si usted usa lentes de contacto la mayoría del tiempo responda las preguntas teniendo en cuenta como se siente cuando esta usando los lentes de contacto
Por favor, marque con una X su opción preferida en cada pregunta	Please, circle your preferred choice in each question	Por favor, circule la mejor eleccion en cada pregunta	Por favor circule la respuesta correcta
A2. ¿Ha notado que a veces se le emborronan las letras del ordenador mientras trabaja con él?	A2. Have you noticed that the letters on the screen become blurry while you're working with your computer?	A2. Ha notado que las letras en la pantalla estan borrosas mientras trabaja en la computadora?	A2. Ha notado que las letras en la pantalla del computador se ven borrosas?
A4. ¿Nota sus ojos cansados durante o después del trabajo con ordenador?	A4. Have you felt your eyes tired during or after working with your computer?	A4. Ha sentido sus ojos cansados durante o despues de trabajar en la computadora?	A4. Alguna vez ha sentido sus ojos cansados después de trabajar en el computador o mientras Trabaja en el computador?
A9. ¿Ha notado que le duelan los ojos en el trabajo?	A9. Did your eyes hurt when working with you computer?	A9 Sus ojos le molestan cuando trabaja en la computadora?	A9. Le duelen los ojos mientras trabaja en el computador?
A17. ¿Ha notado los ojos pesados tras un tiempo con el ordenador?	A17. Have you noticed your eyes heavy after some time working with your computer?	A17. Ha notado sus ojos pesados despues de algun tiempo de haber trabajado en la computadora?	A17. Alguna vez siente sus ojos pesados mientras trabaja en el computador?
A20. ¿Ha notado que cuando usa el ordenador tenga que parpadear mucho?	A20. Did you have to blink a lot while using the computer at work?	A20. Ha tenido que parpadear bastante mientras usa la computadora para trabajar?	A20. Alguna vez siente que tiene que parpadear seguidamente mientras trabaja en el computador?
A21. ¿Ha notado sensación de ardor en sus ojos?	A21. Did you experience burning eyes?	A21. Ha experimentado ardor en los ojos?	A21. Alguna vez le arden los ojos?
A22. ¿Ha notado que, tras un tiempo con el ordenador, tiene que esforzarse para poder conseguir ver bien?	A22. Have you noticed that, after some time working with your computer, you have to strain to see well?	A22. Ha notado que despues de cierto tiempo de trabajar en la computadora usted necesita hacer un esfuerzo para ver bien?	A22. Alguna vez ha notado luego de trabajar en el computador que sus ojos se sienten tensos?
A28. ¿Mientras lee o escribe con su ordenador ¿tiene la sensación de que se ponga bizco?	A28. While you're reading or writing on your computer, did you feel like you're crossing your eyes?	A28. Mientras esta leyendo o escribiendo en la computadora, usted ha sentido que se le cruzan los ojos?	A28. Cuando usa su computador o escribe en su computador siente que sus ojos se cruzan?
A30. ¿Ha notado que cuando pasa mucho tiempo con el ordenador llega un momento en que se acaba viendo las letras dobles?	A30. Have you noticed that, after a lot of time on the computer, the letters appear as a double image?	A30.Ha notado que despues de mucho tiempo en la computadora las imagenes de las letras aparecen doble?	A30.Alguna vez ha notado luego de usar su computador por algún tiempo que las letras se ven doble?
A32. ¿Con que frecuencia ha notado escozor en la vista mientras esta delante del ordenador?	A32. How frequently have you noticed stinging in your eyes while working on the computer?	A32. Cuan frecuente ha notado punzante sus ojos mientras trabaja en la computadora?	A32. Cuan frecuente ha notado que le pican los ojos mientras usa el computador?
A33. Have you noticed that the lights bother you after some time on the computer?	A33. Have you noticed that the lights bother you after some time on the computer?	A33. Ha notado que la luz le molesta despues de cierto tiempo en la computadora?	A33. Alguna vez ha notado que le molestan las luces del ambiente o el techo después de usar el computador?

Instructions: A CONTINUACIÓN, TENIENDO EN CUENTA SUS SENSACIONES DURANTE LAS CUATRO ÚLTIMAS SEMANAS, INDIQUE HASTA QUE PUNTO HA EXPERIMENTADO LAS SIGUIENTES MOLESTIAS:	Now, regarding your experience during the last four working weeks, please indicate to what extent you've felt the following troubles:	Ahora, en relacion a su experiencia durante las ultimas cuatro semanas de trabajo, por favor indique el grado en que ha sentido los siguientes problemas.	Durante las ultimas cuatro semanas de trabajo por favor indique cual ha sido su experiencia
Si usa gafas o lentes de contacto habitualmente en su trabajo, por favor responda a todas las preguntas pensando en cómo se siente cuando sí las lleva puestas	If you normally wear glasses or contact lenses during most of your working hours, answer as if you were wearing them.	Si normalmente usa espejuelos durante la mayoria de las horas de trabajo, responda si los ha estado usando.	Si típicamente usa espejuelos o lentes de contacto por favor responda las siguientes preguntas como si estuviera usando los espejuelos o los lentes de contacto
B7. Lagrimeo	B7. Watery eyes	B7. Ojos Humedos	B7. Le lagrimean los ojos
B8. Ojos Rojos	B8. Eye redness	B8. Ojos Rojos	B8. Tiene los ojos rojos
Instructions: POR FAVOR, DIGA SI LE PARECE CIERTA O FALSA CADA UNA DE LAS SIGUIENTES FRASES	To finish, please indicate to what extent you consider true or false each one of the following statements.	Para terminar, por favor indique en que grado usted considera verdadero o falso cada una de las siguientes declaraciones.	Para concluir, por favor indique si es falso o verdadero
Si usa gafas o lentes de contacto habitualmente en su trabajo, por favor responda a todas las preguntas pensando en cómo se siente cuando sí las lleva puestas	If you normally wear glasses or contact lenses during most of your working hours, answer as if you were wearing them.	Si normalmente usa espejuelos durante la mayoria de las horas de trabajo, responda si los ha estado usando.	Si típicamente usa espejuelos o lentes de contacto por favor responda las siguientes preguntas como si estuviera usando los espejuelos o los lentes de contacto
C16. Al final de la jornada de trabajo noto que me pesan los ojos	C16. At the end of my working day, I feel heavy eyes	C16. Al final del dia yo siento mis ojos pesados	C16. Al final del día del trabajo mis ojos se sienten pesados
C21. Tras un tiempo con el ordenador, noto que tengo que esforzarme para ver bien	C21. After some time at the computer, I have to strain to see well	C21. Despues de algun tiempo en la computadora, yo necesito esforzarme para ver bien.	C21. Después de usar el computador por un tiempo, tengo que esforzarme para ver bien
C23. Durante el trabajo, tengo que cerrar los ojos para aliviar la sequedad que noto en los ojos	C23. While I'm working, I have to close my eyes to relieve eye dryness	C23. Mientras trabajo tengo que cerar mis ojos para mejorar la sequedad.	C23. Mientras estoy trabajando, tengo que cerrar los ojos para aliviar la sequedad
C24. Tras un tiempo con el ordenador, me molestan las luces	C24. After some time at the computer, the lights bother me	C24 Despues de algun tiempo en la computadora las luces me molestan.	C24. Después de algún tiempo en el computador las luces me molestan
**Translation of response categories**			
1. No, nada<br>2. Sí, muy poco<br>3. Sí, un poco<br>4. Sí, moderadamente<br>5. Sí, mucho<br>6. Sí, muchísimo	1. None at all<br>2. Very little<br>3. Little<br>4. A moderate amount<br>5. Much<br>6. Very much	1. No<br>2. Un poco<br>3. poco<br>4. una cantidad moderada<br>5. mucho<br>6. muchisimo	1. Nunca<br>2. Casi Nunca<br>3. Poco<br>4. Más o menos<br>5. Bastante<br>6. Muchas veces
1. Nunca<br>2. Casi nunca<br>3. Poco tiempo<br>4. Parte del tiempo<br>5. Mucho tiempo<br>6. Casi siempre<br>7. Siempre	1. Never<br>2. Almost never<br>3. Seldom<br>4. Ocassionally<br>5. Frequently<br>6. Almost always<br>7. Always	1. Nunca<br>2. Casi nunca<br>3. Rara vez<br>4. Ocasionalmente<br>5. Frecuentemente<br>6. Casi siempre<br>7. Siempre	1. Nunca<br>2. Casi nunca<br>3. Raramente<br>4. Ocasionalmente<br>5. Frecuentemente<br>6. Casi siempre<br>7. Siempre
4. Constantemente<br>3. Frecuentemente<br>2. Raramente<br>1. Nunca	4. Always<br>3. Frequently<br>2. Rarely<br>1. Never	4. Siempre<br>3. Frecuentemente<br>2. Raramente<br>1. Nunca	4. Siempre<br>3. Frecuentemente<br>2. Raramente<br>1. Nunca
1. Nunca<br>2. Casi Nunca<br>3.Unas Pocas Veces<br>4. Varias Veces<br>5. Muchas Veces<br>6. Muchísimas Veces	1. Never<br>2. Almost never<br>3. A few times<br>4. Several times<br>5. Often<br>6. Very Often	1. Nunca<br>2. Casi nunca<br>3. Pocas veces<br>4. Varias veces<br>5. A menudo<br>6. Muy a menudo	1. Nunca<br>2.Casi nunca<br>3. Pocas veces<br>4. Varias veces<br>5. Seguido<br>6. Bien seguido
1.Nada<br>2.Muy Poco<br>3.Un poco<br>4.Moderadamente<br>5.Mucho<br>6.Muchísimo	1.None<br>2.Very little<br>3.Litte<br>4.A moderate amount<br>5.Much<br>6.Very much	1.Ninguno<br>2.Muy poco<br>3.Poco<br>4.Cantidad moderada<br>5.Mucho<br>6.Muchisimo	1.Nunca<br>2.Muy poco<br>3.Poco<br>4.Bastante<br>5.Mucho<br>6.SIempre
1. Bastante falsa<br>2. Totalmente falsa<br>3. Bastante cierta<br>4. Totalmente cierta	1. Quite false<br>2. Completely false<br>3. Quite true<br>4. Completely true	1. Absolutamente falso<br>2 Completamente falso<br>3. Absolutamente verdadero<br>4. Completamente verdadero	1. Falso<br>2. Completamente falso<br>3. Verdadero<br>4. Completamente verdadero

Item ID	Item Descriptor	Missing responses (%)	Measure	Infit MnSQ	Outfit MnSQ	Pt.Bis
	THE QUESTIONS THAT FOLLOW ASK ABOUT HOW YOU FELT OVER THE PAST FOUR WEEKS WHILE AT WORK.*: *If you normally wear glasses or contact lenses during most working hours, please describe how you felt while wearing this correction.					
	While you were working on the computer for a while…					
**A2**	***Did the letters on the screen become blurry?***	0	1.39	0.95	1.28	0.52
**A4**	***Did your eyes become tired?***	0	-2.46	1.15	1.21	0.58
**A9**	***Did your eyes hurt?***	0	0.08	0.92	0.92	0.64
**A20**	***Did you have to blink more than usual?***	0	-1.1	0.77	0.79	0.77
**A21**	***Did your eyes burn?***	0	-0.2	1.52	1.51	0.47
**A22**	***Did you have to strain to see well?***	0	-1.3	0.86	0.8	0.72
**A28**	***Did you feel like you were crossing your eyes?***	0	0.73	0.74	0.64	0.69
**A30**	***Did the letters appear double?***	0	0.55	0.83	0.72	0.61
**A32**	***Did your eyes sting?***	0	3.06	1.16	0.77	0.14
	After working on the computer for a while…					
**A17**	***Did your eyes become heavy?***	0	0.67	0.69	0.67	0.72
**A33**	***After working on the computer for a while did lights bother you?***	0	-1.13	0.97	1.02	0.62
	OVER THE PAST FOUR WEEKS WHILE AT WORK, PLEASE INDICATE TO WHAT EXTENT YOU HAVE EXPERIENCED ANY OF THE FOLLOWING:					
**B7**	***Watery Eyes***	0	-0.15	1.13	1.15	0.4
**B8**	***Eye redness***	2	0.73	1.05	1.37	0.45
	PLEASE INDICATE TO WHAT EXTENT YOU AGREE OR DISAGREE EACH ONE OF THE FOLLOWING STATEMENTS:					
**C16**	***At the end of my working day, my eyes feel heavy***	0	-0.73	1.11	1.03	0.61
**C21**	***After working at the computer, I have to strain to see well***	0	0.19	1.1	1.1	0.54
**C23**	***I have to shut my eyes hard to relieve dryness when using these devices***	0	-0.83	0.95	0.85	0.67
**C24**	***After some time at the computer, lights bother me***	0	0.25	0.25	-0.02	0.54

Table. Item statistics emerging from the Rasch-analysis pretest. Results are ordered by Item Id (left column). Measure= Item difficulty (in logits); MnSQ= Mean Square; Pt. Bis= Point bi-serial correlation
