# Supplementary material for: English version of the Computer Vision Symptom Scale (CVSS17): Translation and Rasch analysis-based cultural adaptation
Source: PLoS One. 2025 Apr 16;20(4):e0316936. doi: 10.1371/journal.pone.0316936 (PMC12002468; doi:10.1371/journal.pone.0316936)
Supplement: S2 File — PDF version of the CVSS17ENG, for those interested in distributing it as a hard copy. (PDF) [file pone.0316936.s002.pdf]

COMPUTER VISION SYMPTOM SCALE (CVSS17)

Final, cross-culturally adapted, English Version

THE QUESTIONS THAT FOLLOW ASK ABOUT HOW YOU FELT OVER THE PAST FOUR WEEKS WHILE AT WORK.*:
*If you normally wear glasses or contact lenses during most working hours, please describe how you felt while wearing this correction.

While working on the computer for a while (please circle the appropriate response):

A2. Did the letters on the screen become blurry?

1. Never 2. Very Little 3. Little
4. A moderate amount 5. Much 6. Very Much

A4. Did your eyes become tired?

1. Never 2. Almost Never 3. Seldom 4. Occasionally
5. Frequently 6. Almost Always 7. Always

A9. Did your eyes hurt?

1. Never 2. Rarely 3. Frequently 4. Constantly

A17. After working on the computer for a while did your eyes become heavy?

1. Never 2. Rarely 3. Frequently 4. Constantly

A20. Did you have to blink more than usual?

1. Never 2. Rarely 3. Frequently 4. Constantly

A21. Did your eyes burn?

1. Never 2. Rarely 3. Frequently 4. Constantly

A22. Did you have to strain to see well?

1. Not at all 2. Very little 3. A Little

4. A moderate amount 5. Much 6. Very Much

A28. Did you feel like you were crossing your eyes?

1. Never 2. Rarely 3. Frequently 4. Constantly

A30. Did the letters appear double?

1. Not at all 2. Very little 3. A Little

4. A moderate amount 5. Much 6. Very Much

A32. Did your eyes sting?

1. Never 2. Rarely 3. Frequently 4. Constantly

A33. After working on the computer for a while did lights bother you?

1. Never 2. Almost never 3. A few times
4. Several times 5. Often 6. Very often

COMPUTER VISION SYMPTOM SCALE (CVSS17)

Final, cross-culturally adapted, English Version

OVER THE PAST FOUR WEEKS WHILE AT WORK, PLEASE INDICATE TO WHAT EXTENT YOU HAVE EXPERIENCED ANY OF THE FOLLOWING:

	1. None	2. Very little	3. Little	4. A moderate amount	5. Much	6. Very much
**B7. Watery Eyes**						
**B8. Eye redness**						

TO FINISH, PLEASE INDICATE TO WHAT EXTENT YOU AGREE OR DISAGREE EACH ONE OF THE FOLLOWING STATEMENTS*:
*If you normally wear glasses or contact lenses during most of your working hours, answer as if you were wearing them.

C16. At the end of my working day, my eyes feel heavy

1. Strongly Disagree
2. Slightly Disagree
3. Slightly Agree
4. Strongly Agree

C21. After working at the computer, I have to strain to see well

1. Strongly Disagree
2. Slightly Disagree
3. Slightly Agree
4. Strongly Agree

C23. I have to shut my eyes hard to relieve dryness when using these devices

1. Strongly Disagree
2. Slightly Disagree
3. Slightly Agree
4. Strongly Agree

C24. After some time at the computer, lights bother me

1. Strongly Disagree
2. Slightly Disagree
3. Slightly Agree
4. Strongly Agree

Note: This version replaces the preliminary, self-developed English version provided in the CVSS17 Spanish version development paper (https://doi.org/10.1167/iovs.13-13818). Clinicians and researchers interested in administering the English version of the CVSS17 must use the current validated version.
