# Supplementary material for: English version of the Computer Vision Symptom Scale (CVSS17): Translation and Rasch analysis-based cultural adaptation
Source: PLoS One. 2025 Apr 16;20(4):e0316936. doi: 10.1371/journal.pone.0316936 (PMC12002468; doi:10.1371/journal.pone.0316936)
Supplement: S1 Table — Each CVSS17ENG item is identified by a capital letter and a one- or two-digit number. Additionally, each response option for each item is identified by a number. This table is necessary to determine the score that each subject gets for each question when using the hard-copy version (respondents are automatically scored in the online version). For example, if a respondent chooses option 6 for the first question, they will receive three points; choosing option 3 for item A30 gets one point. The final score for each subject is obtained using the formula at the bottom of the table, both in CVSS17 points and in logits. (PDF) [file pone.0316936.s003.pdf]

	Response Option						
**Ítem Id.**	***1***	***2***	***3***	***4***	***5***	***6***	***7***
A2	1	1	2	2	3	3	
A4	1	1	2	2	3	3	3
A9	1	2	3	4			
A17	1	2	3	4			
A20	1	2	3	4			
A21	1	2	3	3			
A22	1	1	2	2	3	3	
A28	1	2	3	3			
A30	1	1	1	2	2	2	
A32	1	2	3	4			
A33	1	2	2	3	3	3	
B7	1	1	2	2	2	2	
B8	1	1	2	2	3	3	
C16	1	1	2	3			
C21	1	1	2	3			
C23	1	1	2	3			
C24	1	1	2	3			

CVSS17 Score = [(Sum of scores) x 17] / (number of valid responses)

CVSS17 Measure (logits)= (0.2615 x CVSS17 Score) - 9.1838
