# Supplementary material for: English version of the Computer Vision Symptom Scale (CVSS17): Translation and Rasch analysis-based cultural adaptation
Source: PLoS One. 2025 Apr 16;20(4):e0316936. doi: 10.1371/journal.pone.0316936 (PMC12002468; doi:10.1371/journal.pone.0316936)
Supplement: Attachment — Submitted filename: Response to reviewers.docx [file pone.0316936.s005.docx]

Response to Journal requirements:

1. When submitting your revision, we need you to address these additional requirements.

Please ensure that your manuscript meets PLOS ONE's style requirements, including those for file naming. The PLOS ONE style templates can be found at <https://journals.plos.org/plosone/s/file?id=wjVg/PLOSOne_formatting_sample_main_body.pdf>and <https://journals.plos.org/plosone/s/file?id=ba62/PLOSOne_formatting_sample_title_authors_affiliations.pdf>.

*According to your advice, we revised file naming and addressed some minor formatting issues, such as changing parentheses to brackets in the references.*

2. Thank you for stating the following in the Acknowledgments Section of your manuscript:

[This study was supported by the Instituto de Salud Carlos III through Project “PI18/00374” (co-funded by European

Regional Development Fund “A way to make Europe”).]

We note that you have provided funding information that is not currently declared in your Funding Statement.

However, funding information should not appear in the Acknowledgments section or other areas of your manuscript.

We will only publish funding information present in the Funding Statement section of the online submission form. Please remove any funding-related text from the manuscript and let us know how you would like to update your Funding Statement. Currently, your Funding Statement reads as follows:

[BA, AB, MGP and RS received a grant by the Instituto de Salud Carlos III (<https://www.isciii.es/Paginas/Inicio.aspx>) through Project “PI18/00374” (co-funded by European Regional Development Fund “A way to make

Europe”). The funders didn't play any role in the study design, data collection and analysis, decision to publish, or preparation of the manuscript]

Please include your amended statements within your cover letter; we will change the online submission form on your behalf.

*Thank you for your advice. We have omitted this information from the acknowledgements section and are providing a new Funding Statement along with the cover letter. The new Funding Statement remains at follows: “This study was supported by a grant by the Instituto de Salud Carlos III (https://www.isciii.es/Paginas/Inicio.aspx) through Project “PI18/00374” (co-funded by the European Regional Development Fund “A way to make Europe”). The funders didn't play any role in the study design, data collection and analysis, decision to publish, or preparation of the manuscript”*

3. Thank you for uploading your study's underlying data set. Unfortunately, the repository you have noted in your DataAvailability statement does not qualify as an acceptable data repository according to PLOS's standards.

At this time, please upload the minimal data set necessary to replicate your study's findings to a stable, public repository (such as figshare or Dryad) and provide us with the relevant URLs, DOIs, or accession numbers that may be used to access these data. For a list of recommended repositories and additional information on PLOS standards for data deposition, please see [https://journals.plos.org/plosone/s/recommended-repositories.](https://journals.plos.org/plosone/s/recommended-repositories)

*Thank you. According to your advice, we’ve uploaded to FigShare the minimal dataset necessary to replicate our study findings. The new dataset’s DOI (10.6084/m9.figshare.25909171) is provided in the corresponding submission section.*

4. Please include captions for your Supporting Information files at the end of your manuscript, and update any in-textcitations to match accordingly. Please see our Supporting Information guidelines for more information: <http://journals.plos.org/plosone/s/supporting-information>.

*Thank you. We missed including it in the first version, so in the new version of the manuscript, a list of the Supporting Information captions is provided at the end of the manuscript in a section titled 'Supporting Information'*

1. Response to review Comments to the Author

Please use the space provided to explain your answers to the questions above. You may also include additional comments for the author, including concerns about dual publication, research ethics, or publication ethics. (Please upload your review as an attachment if it exceeds 20,000 characters)

Reviewer #1: comments to improve the paper.

- provide more details on the translation and cross-cultural adaptation process. specifically, describe the steps taken to ensure cultural equivalence and appropriateness of the translated items.

*Thank you for your suggestion. Based on it we have provided this information in lines 121-127*

- clarify the inclusion and exclusion criteria for the participants in the two stages of the study.

*According to your recommendation, we’ve clarified the inclusion criteria for stage 1 (lines 149-160) and for stage 2 (lines 177-187)*

- provide more information on the recruitment process, especially for the online panel used in stage 2.

*Thank you for your suggestion. Based on it we have provided more information about the online panel used in stage 2. (lines 169-174)*

- Justify sample size for each

*Thank you for your feedback. Regarding sample size justification:*

*The justification for sample size used in Stage 1 is described in lines 146-150*

*Justification for sample size used in the validation of the CVSS17ENG and in DIF analysis is described in lines 189-191*

*Justification for sample size used in the convergent validity and test-retest reliability assessment is displayed in lines 198-202.*

.

statistical analyses need major rework for explanations.

- the rationale for choosing the partial credit model (pcm) over the rating scale model (rsm) for rasch analysis should be explained in more detail.

*Thank you for your suggestion. Based on your feedback, we have provided a more detailed explanation (lines 230-241) of the rationale for choosing the Partial Credit Model (PCM) over the Rating Scale Model (RSM).*

- I am confused if this is irt or mirt?

*Thank you for your observation. In the revised manuscript (lines 225-228), we have clarified that we used a unidimensional Item Response Theory (IRT) model.*

- the method used to assess unidimensionality should be described more clearly. the authors mention using the disattenuated correlation coefficient, but it would be helpful to provide a brief explanation of this approach. Use updated refs.

*Thank you. Following your recommendation, we have added a brief explanation (lines 258-262) about the use of the disattenuated correlation coefficient for assessing unidimensionality when the PCA analysis suggests multidimensionality.*

- the authors should consider providing more details on the assessment of differential item functioning (dif) and the interpretation of the dif contrast values. I am lost in present manuscript.

*Thank you for your observation. Based on your feedback, we have included I the new manuscript more details abaut the DIF assessment and its interpretation (lines 273-283)*

- the interpretation of the person separation index (psi) and the levels of performance should be clarified for readers who may be less familiar with rasch analysis.

*According to your suggestion, we have included in the new manuscript a comment in line 264, and a brief section in the discussion (lines 437-455).*

- the authors should explain the criteria used to determine acceptable fit statistics (e.g., the recommended range for infit and outfit mean square values).

*Based on your feedback, the recommended range for infit and outfit mean square values are now described in the methods section of the manuscript (lines 248-250).*

- provide more information on the methods used to assess convergent validity, specifically the rationale for choosing the visual discomfort scale (vds) and the ocular comfort index (oci) as comparison measures.

*According to your observation, we discuss it in the new manuscript (482-489).*

- the authors should consider presenting the correlation coefficients between cvss17eng and vds/oci with their corresponding confidence intervals.

*Thank you for the suggestion. According to it, we have included a new table (Table 5) in the manuscript (lines 411-415) that summarizes the results of the convergent validity assessment, including the confidence intervals of the correlation coefficients.*

results and discussion

- it would be helpful to include a table summarizing the demographic characteristics of the participants in both stages of the study.

*Based on your feedback, we have modified Table 3 and know it provides the demographic characteristics of all the samples used in the study*

- the results for the convergent validity assessment with vds and oci could be presented more clearly, perhaps with additional figures or tables. better visually.

*According to your suggestion, we have included a new table (Table 5) in the manuscript (lines 411-415) that summarizes the results of the convergent validity assessment and completes the information displayed in Fig 2 and Fig 4. Thank you*

- the authors should consider discussing the clinical significance or implications of the observed differences in dif between the english and spanish versions.

*Thank you very much for your suggestions to improve the paper, according to your feedback we’ve added a paragraph in the discussion (lines 498-507) about the implications of the DIF analysis results.*
